# Spatial and Temporal Adaptations of Lowland Tapirs (*Tapirus terrestris*) to Environmental and Anthropogenic Impacts

**DOI:** 10.3390/life13010066

**Published:** 2022-12-25

**Authors:** Kathrin Burs, Lydia Möcklinghoff, Marinez Isaac Marques, Karl-L. Schuchmann

**Affiliations:** 1 Computational Bioacoustics Research Unit (CO.BRA), National Institute for Science and Technology in Wetlands (INAU), Federal University of Mato Grosso (UFMT), Fernando Correa da Costa Av. 2367, Cuiabá 78060-900, MT, Brazil; 2 Section of Ornithology, Zoological Research Museum Alexander Koenig (ZFMK), Adenauerallee 160, 53113 Bonn, Germany; 3 Postgraduate Program in Zoology, Institute of Biosciences, Federal University of Mato Grosso, Cuiabá 78060-900, MT, Brazil

**Keywords:** *Tapirus terrestris*, Pantanal, camera traps, anthropogenic impact, environmental impact, spatial adaptation, temporal adaptation

## Abstract

The Pantanal is one of the most conserved wetland ecosystems in Brazil and a hotspot for biodiversity. Over the last decades intensification of human activities has become a major threat to the stability of the unique landscape. To establish effective conservation actions, it is essential to understand how species respond to anthropogenic and environmental regional factors. Here, data from two multiannual camera trap studies, one in the northern Pantanal and one in the southern Pantanal, were used to investigate the effects of habitat characteristics, seasons, and human interactions on the spatial and temporal patterns of lowland tapirs (*Tapirus terrestris)*. Between 2010 and 2017, camera traps were repeatedly placed in consistent grids covering protected areas and areas with cattle-ranching and tourism. Data were analyzed using generalized linear mixed models and circular statistics. Activity patterns were similar and predominantly nocturnal in both areas, but tapirs indicated avoidance toward settlements and cattle and indicated habitat preferences only in the northern study area with less anthropogenic activities. The present study suggests that both environmental and anthropogenic factors can affect the species’ spatial and temporal behavior, but tapirs show varying responses across regions and gradients of disturbance. The results indicate that adapting avoidance strategies might be more likely and effective in areas with low human pressure and sufficient protected areas as alternatives.

## 1. Introduction

Worldwide anthropogenic pressure, especially habitat degradation and conversion, negatively affects species abundance, occurrence, richness and movement behavior [1,2,3], and habitat loss, mainly driven by intensification of agriculture, is by far the most significant threat to mammal species [4].

Human activities such as cattle ranching and the associated habitat alteration and forage competition have well-documented negative effects on the presence, abundance and richness of mammals [5,6,7,8]. Additionally, generally considered biodiversity-compatible human activities such as wildlife-focused ecotourism or outdoor recreation [9,10,11] can have negative effects on mammal species presence or diversity and might cause spatial avoidance behavior in certain mammal species when operated at a larger scale [12,13].

Human disturbance can also have more subtle effects and has been shown to cause shifts in the activity of mammal species worldwide, with increased nocturnality in areas or time periods with high disturbance [14]. Where large suitable habitats are missing spatial avoidance is not always an option and needs to be traded off with resource availability [15,16]. Temporal adaptation appears to be an effective strategy to coexist with humans and has been observed in different mammal species as a response to hunting, facilitated human access to an area or settlement, or cattle or human presence in agricultural areas [17,18,19,20,21]. In protected areas with low pressure, however, environmental factors might play a more important role in mammal species activity than human disturbance factors [22].

The responses to human pressure are species-specific and have been shown to be associated with the ecological and life history traits of a species and vary within species depending on the type and intensity of pressure [23,24,25]. As human disturbance can be expected to become worse over the next decades, it is critical to understand the impact of anthropogenic factors while accounting for environmental factors. This can be especially important for vulnerable species such as lowland tapirs, which play a crucial role in the maintenance of Neotropical forests due to their function as seed dispersers [26]. Throughout the species range, habitat loss, illegal hunting, roadkill, competition with livestock and isolation of populations are considered major threats, and lowland tapirs are currently listed as vulnerable, with a decreasing population [27,28].

To date, little is known about the impact of human activities on lowland tapirs in the Pantanal [29,30,31,32,33]. The region is considered an important stronghold for the tapir population [27], and compared to other Brazilian biomes, the Pantanal is still in a rather pristine condition, with up to 89.55% natural vegetation [34,35,36,37]. Natural flood pulses with seasonal droughts and floods of varying intensities [38,39,40] limit the agricultural use of the area [36]. Cattle ranching has been the major source of income in the region for more than three centuries [41], but traditional cattle management with the movement of herds among natural pastures is generally considered to have only a low environmental impact in the Pantanal [42,43,44,45]. Recently, ecotourism based on wildlife observation has become another important economic factor in the region [46]. Studies suggest that when operated at a small scale, both sectors have little effect on mammal communities in the Pantanal and can support sustainable development [29].

Over the last decades, however, the intensification of agricultural activities, deforestation, changes in the flood regime, fires, and climate change have increasingly threatened the Pantanal ecosystem [37,41,44,47,48,49,50,51]. The conversion of natural vegetation to human-use areas substantially increased between 1976 and 2017, and if trends continue, 29% of the Pantanal area might be converted by 2050 [36]. Recently, the Pantanal has been subject to prolonged droughts and historically unprecedented fire events burning 30% of the biome´s area [52,53]. Due to climate change, severe periods of drought are expected to become more frequent, affecting flooding dynamics and ecosystem functioning [51].

The future of lowland tapirs in the Pantanal depends on their ability to respond to these rapid environmental changes. Given their wide distribution across various regions [54], the species seems to be able to adapt to almost every habitat in South America. They are generally considered to have a strong association with water bodies and inundated, moist habitats [55,56,57], but they were also shown to adapt well to seasonal dry conditions [58]. Several studies indicate that forests, especially with a high density of palm trees, are important forage sources and resting places for the species [57,59,60,61,62]. In the dense landscape mosaic of the Pantanal, lowland tapirs exhibit a rather flexible habitat use including forest, grassland and savanna [29,63,64,65,66,67]. The species also uses degraded habitats and anthropologically used areas, and is present in disturbed and secondary forests, tree plantations and agricultural land [28,57,68]. Recent studies indicate that lowland tapirs show little response to human or cattle interference [29,30], but also that the species might lack differentiated behavior in areas with varying disturbance, which could risk human-altered habitats becoming ecological traps [31]. In contrast, there is also evidence that lowland tapirs respond spatially to human disturbance [13,62,69,70,71], but several studies suggest that natural factors play a more important role in the activity and occurrence of lowland tapirs than human disturbances [32,65,72,73]. Changes in activity have been observed mainly with relation to habitat, temperature, season or availability of forage sources [32,57,74,75,76,77].

Despite the growing numbers of researchers studying lowland tapirs [5], there is still a lack of long-term studies in the Pantanal. Existing studies on the species behavior cover periods of up to 2 years [32,64,65,78], but were often only conducted over several months [29,30,33,63,66,67,73]. Currently, Medici et al. [31] provides the longest data collection, covering the impressive period of 22 years. 

Here, camera trap data from two multiannual studies conducted between 2010 and 2017 were used to investigate the potential impact of anthropogenic and environmental factors on lowland tapirs at two sites with varying intensities of human disturbance in the southern and northern Pantanal. The two major human activities in the region were considered and represented by cattle presence, distance to tourist trails and roads, and settlement. To account for environmental aspects, habitat type, distance to water bodies and seasonal period were considered. First, the effects of the chosen factors on the number of tapirs in each study area were assessed. Then, tapir activity patterns and levels were estimated and compared between both study areas, and the effects of the factors on the probability of nocturnal activity were assessed.

By incorporating both tapir count and activity data and investigating areas with varying human pressure, the present study aims to better understand the adaptive strategies that lowland tapirs might use to thrive in a region with centuries of human land use tradition.

## 2. Materials and Methods

### 2.1. Study Areas

**Figure 1:**
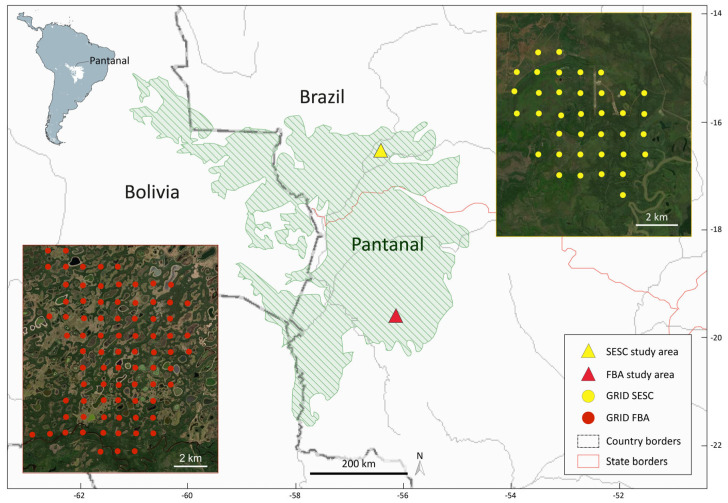
Geographical location of study areas in the northern (SESC) and southern (FBA) Pantanal with grid shapes and camera trap stations (dots) (SESC = 37, FBA = 80). Map source: GGIS 3.12.1; Pantanal shape file source: Bioscience, An Ecoregions-Based Approach to Protecting Half the Terrestrial Realm.

Data collection was conducted in two separate study areas: Fazenda Barranco Alto (FBA) in the southern Pantanal of Mato Grosso do Sul (19°34′40″ S 56°09′08″ W) and Parque Sesc Baía das Pedras (SESC) in the northeastern Pantanal of Mato Grosso (16°49′88″ S 56°41′30″ W) (Figure 1).

Fazenda Barranco Alto is located in the Rio Negro Basin at the southeastern outskirts of the Nhecolândia subregion of the Brazilian Pantanal. It is an 11,000-hectare-sized, traditionally managed cattle ranch with approximately 2000 heads of cattle and a small lodge with 16 beds for ecotourism. Tourists use the area between 6 and 11 am and between 15 and 19 pm. Approximately 50% of the area is generally accessible for tourism [79]. Activities concentrate on horseback riding, boat tours, nature walks and safaris by car. The river “Rio Negro” flows through the farm area. The core study area covered the 7355 hectare area north of the river, where farmhouses and infrastructure are also located. 

Parque Baía das Pedras is located in the Cuiabá River floodplain and an approximately 4200 hectare unit of the SESC nature reserve. Tourism activities within this area concentrate on day visitors, who take part in nature walks and horseback riding during the late morning between 9 and 12 in approximately 10% of the area. No cattle are kept on the property, but during the study, small groups of cattle that accidentally entered from neighboring farms were sighted and recorded by camera traps.

Influenced by the adjacent Cerrado, Chaco, and Amazon biome [80,81], the Pantanal is characterized by a diverse mosaic of mesic, xeric and hydric habitats, which also characterize the study areas. Forests and savanna patches intersperse with freshwater bodies. In the study area in the southern Pantanal, there are also more than one hundred soda lakes, the so-called “Salinas”, present [82]. The northern Pantanal is generally susceptible to greater flood fluctuations and more pronounced dry periods than the southern region [83], and holds a higher proportion of swampy and floodable habitats [37,84].

### 2.2. Data Collection

In both study areas, camera trap sites were established in a regular grid, maintaining a 1 km distance (FBA +/−20 m, SESC +/−60 m) between each site (Figure 1). The grids were digitally generated using Hawth’s Tools (vers. 3.27) [85] extension with ArcGIS 9.3 (©ESRI 2009). At SESC, a total of 37 camera trap sites were established. At FBA, a total of 80 camera trap sites were established (Figure 1). Each camera trap site consisted of one camera trap. The predefined camera trap sites remained the same during the whole study period. Between 2010 and 2017, a total of 14 field surveys were conducted. Field surveys covered one or both seasonal periods of the Pantanal to collect data from each camera trap site during times of drought and flooding. During each field survey, camera traps were successively placed along the grid in smaller sections. Placement of the sections was planned according to the expected changes in water levels to sample as many sites as possible during each field survey and all sites during both seasonal periods. Camera traps remained at one site for a minimum of 14 (SESC) and 7 (FBA) consecutive nights and days (= one sample) and were then relocated to the next site. Camera traps were active 24 h and operated using a passive infrared-triggered system. At FBA, all accessible camera trap sites were sampled once per field survey. At SESC, the smaller grid size, higher number of camera traps and longer field surveys allowed the repeated placement of grid sections during different times within a field survey.

At SESC, data were collected during 4 field surveys lasting 3 to 6 months between 2015 and 2017. To collect data, 5 camera trap models were used (RECONYX PC800, RECONYX HC600, Bushnell Trophy Cam HD Aggressor, Bushnell Trophy Cam HD2012, UWAY VH400HD). A total of 4862 trap days during 255 samples at 37 sites were conducted. Between 3 and 13 samples per site and a total of 48 to 252 trap days per site were obtained. Due to malfunctions and the influence of the flood regime, the number of trap days camera traps remained active per sample varied between 7 and 75.

At FBA, data were collected during 10 field surveys of 3 months each between 2010 and 2017. To collect data, one camera trap model was used (RECONYX HC500). A total of 4977 trap days during 566 samples at 80 sites were conducted. Between 3 and 10 samples per site and a total of 24 to 90 trap days per site were obtained. The number of trap days camera traps remained active per sample varied between 3 and 23. 

### 2.3. Camera Trap Data Analysis

Only independent records of tapirs were counted as valid. An independent record was defined as (1) consecutive images of different individuals, (2) consecutive images of individuals taken more than 0.5 h apart and (3) nonconsecutive images of individuals [86]. Tapirs can have distinct marks, including white spots and stripes on the stomach or legs, white markings at the ears, scars or torn ears, and can often be determined by sex from photographs [87,88,89,90]. The distinction of two consecutive individuals in the present study focused on sex and juvenile pattern, as these were the easiest visible features in both data sets and tapirs are primary solitary, aside from a courting pair or a female and her offspring [91]. 

With each record, the camera traps stored information about time, date, temperature and moon phase.

### 2.4. Potentially Impacting Factors

To assess the potential impact of anthropogenic and environmental factors in both study areas, (1) the occurring habitat at each camera trap site was determined and roughly categorized into forest or savanna habitat [81], (2) the presence or absence of cattle at each camera trap site was estimated based on camera trap data, (3) each sample was categorized as taken during the dry period (April to September) or rainy period (October to March) [92], and the linear distance of each camera trap site to the next (4) dirt road or trail used for tourism, (5) small settlement, (6) permanent freshwater lake, and (7) saltwater lake (only FBA) was measured. All distance measures were conducted using QGIS (vers. 3.12.) (QGIS Development Team 2020).

### 2.5. Statistical Analysis

All statistical analyses were conducted in R (vers. 3.6.2, The R Foundation for Statistical Computing 2019) [93].

#### 2.5.1. Count Data Analysis

For each camera trap site, the number of tapirs recorded per trap date was estimated. To assess the effect of the determined factors in each study area, negative binomial generalized linear mixed models (GLMMS) were developed since the count data were non-normal and overdispersed. For the analysis, the *glmmTMB* package (vers 3.6.2) [94] was used. In each model, the camera trap site ID and—sample ID were included as random effects to account for nondependence of samples from the same camera trap site and records from the same continuous sample. The categorical factors *habitat*, *period* and *cattle* were mean centered, and distance measurements were standardized into z scores to facilitate comparison of model estimates [95].

For each study area, a model including all determined factors was fit, but alternative candidate models that might better explain the variation in the number of tapirs in the study areas were considered. Short-term studies from the same regions suggest that habitat type or cover, distance to water bodies or cattle-ranching and tourism have little effect on species abundance or occupancy [29,67], and lowland tapirs are reported as semi-aquatic and well-adapted to floodable habitats [55]. Given this knowledge, all factors could or could not individually or in combination affect the species; thus, all possible combinations of the factors (without interactions) were modeled. This resulted in a candidate set of 64 models for SESC and 128 for FBA (as the salt lakes were an additional factor). Both random effects were included in all models. To avoid collinearity among factors, the variance inflation factor (VIF) of the full additive model was tested using the *performance* package (vers.0.7.3) [96], with a VIF less than 5 indicating a low correlation [97]. The support from the data for each model was examined using the Akaike Information Criterion (AIC). Models were ranked based on AIC and in case top-ranked models indicated similar AIC (≤2 AIC), model averaging was employed [98]. Model selection and averaging were performed using the *MuMIn* package (vers.1.43.17) [99]. The significance of the effect of a factor on tapir counts was determined using the model-averaged parameter estimates and their 95% confidence intervals (CIs). Factors for 95% CIs that did not include zero were considered significant [95].

#### 2.5.2. Activity Data Analysis

Activity level (proportion of hours per day spent active), activity pattern (distribution of the activity throughout the day), and the influence of the determined factors on activity were estimated for each study area using time-of-detection data provided with each camera trap record. Activity patterns and activity levels were also compared between the study areas to investigate whether the species’ activity varied between the two areas or regions.

To estimate the activity level, a flexible circular kernel distribution was fit using the *activity* package (vers.1.3.1) [100]. For each of the estimates, a bootstrap with 1000 resampling events was conducted. The activity levels of both study areas were then compared using the Wald test [101].

To estimate and compare the activity patterns of both study areas, circular kernel density functions were fit and the coefficient of overlapping was calculated using the *overlap* package (vers. 0.3.3). The coefficient of overlap ranges from 0 (no overlap) to 1 (complete overlap, identical activity pattern). The estimator Δ_4_, which is suitable for sample sizes greater than 75, was used. The 95% confidence intervals for Δ_4_ were calculated from 10,000 bootstrap samples [102,103]. Watson’s two-sample test was then conducted to determine whether tapir activity patterns were significantly different in the two study areas using the *CircStats* package (vers. 0.2-6) [104].

To investigate whether tapirs´ diurnal or nocturnal activity within each study area was affected by the determined factors, GLMMs with binomial distribution were conducted. First, all records were classified as either diurnal (6:00 to 17:59 h) or nocturnal (18:00 to 05:59 h) to estimate the probability of nocturnal activity. Second, all diurnal records were excluded and the nocturnal records split into hours with high or low activity (above or below the average activity during this phase) to estimate the probability of high nocturnal activity. The second analysis was then repeated considering a less strict classification of nocturnal (17:00 to 6:59 h) to include crepuscular hours.

For each analysis, all possible factor combinations were modeled. Model selection and averaging was performed following the procedure described for the count data analysis using a variant of the AIC, the AICc, which is more suitable for small sample sizes [105].

## 3. Results

### 3.1. Trapping Success

At SESC, a total of 338 tapir records (7 records per 100 trap days) were obtained. The species was recorded at 31 (84%) camera trap sites and during 111 (44%) samples. At FBA, a total of 308 records (6 records per 100 trap days) were obtained. The species was recorded at 70 (88%) camera trap sites and during 173 (31%) samples.

### 3.2. Number of Tapirs

**Figure 2:**
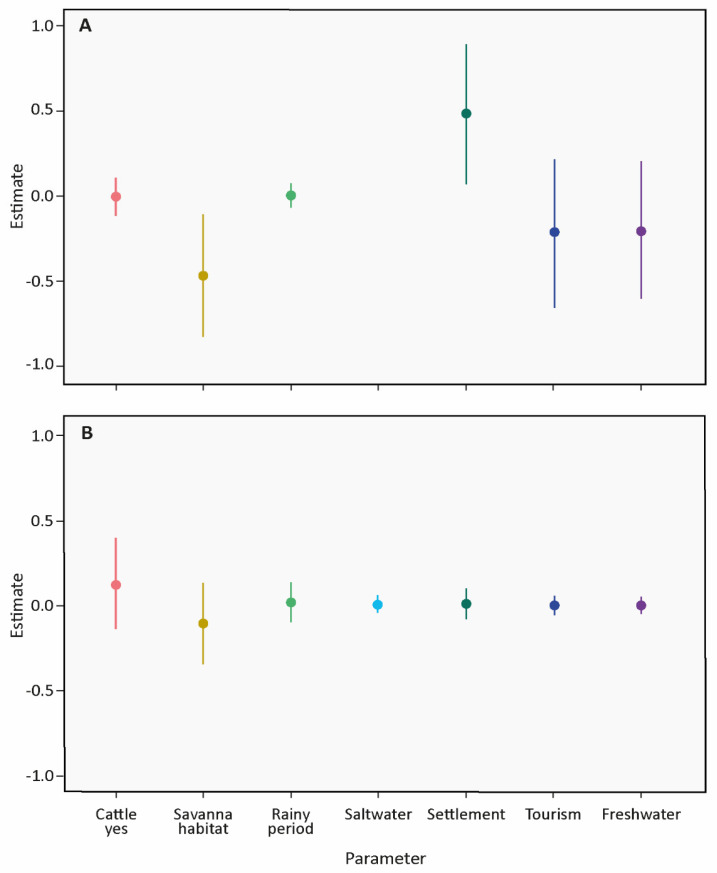
Averaged parameter estimates and corresponding 95% confidence intervals (CI) of generalized linear mixed models (GLMMs) assessing the effect of distinct factors on the number of tapirs in the two study areas (**A**) SESC and (**B**) FBA. Factors included in the analysis: habitat at camera trap site (forest, savanna); cattle presence at camera trap site (no, yes); period during the camera trap sample (dry, rainy); distance of camera trap site to the next freshwater lake, settlement or saltwater lake (only FBA); and dirt roads or trails used for tourism. Random effects, camera trap site ID and sample ID were included in all models.

For SESC, 6 models indicated similar AIC values and were selected for model averaging; for FBA, 11 models were averaged. All determined factors were included in the best model sets (Table 1). The results suggest that at SESC, the number of tapirs is significantly affected only by type of habitat and distance to settlement. The species used savanna habitats less, and the number of tapirs increased as the distance to settlement increased. Period, cattle or distance to tourism or freshwater bodies had no effect on the number of tapirs (Figure 2A, Table S1). At FBA, none of the factors had a significant effect on the number of tapirs (Figure 2B, Table S1). The VIFs of the factors in the fitted full models ranged between 1.01 and 2.03 (SE = 1.01–1.43) for SESC and 1.02 and 1.80 (SE = 1.01–1.34) for FBA, suggesting low correlation among factors. 

### 3.3. Activity of Tapirs

**Figure 3:**
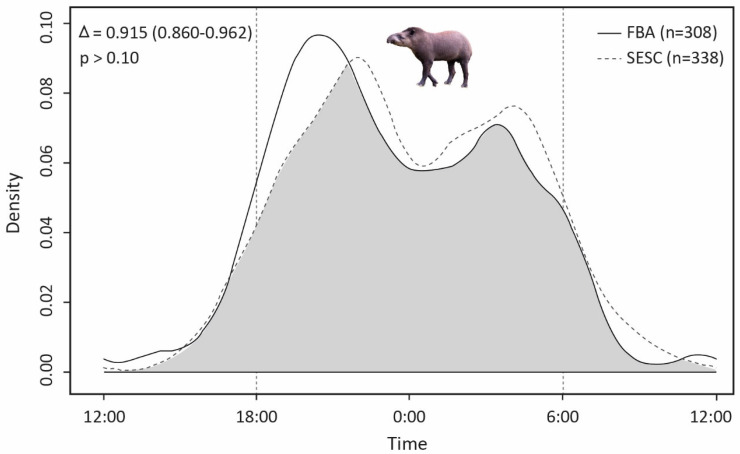
Overlap of activity patterns of tapirs in the two study areas (grey) with overlap coefficient Δ_4_, corresponding confidence intervals, and results of the Watson´s two-sample test (top left).

Activity levels were similar in both study areas (difference: −0.031; Wald test: 0.335 *p*-value: 0.563), with a level of 0.462 (SE = 0.039) for SESC and 0.431 (SE = 0.038) for FBA. The activity patterns of both areas indicated a very high overlap and did not significantly differ (Figure 3). In both study areas, tapirs were predominantly nocturnal and largely inactive between approximately between 7 am and 16 pm, with the lowest activity around noon. In both study areas, a strong increase in activity after approximately 17 pm was observed, reaching its peak of activity around 22 pm at SESC and between 20 and 21 pm at FBA. In both areas, activity declined toward midnight and increased after 1 am or 2 am, reaching a smaller peak at approximately 4 am and between 3 and 4 am, respectively. After 4 am, the activity decreased in both areas. 

**Figure 4:**
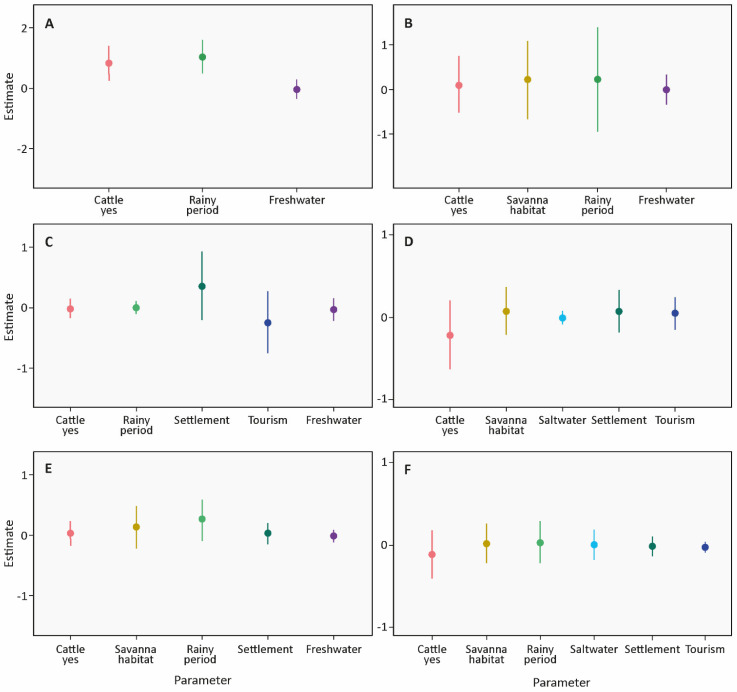
Averaged parameter estimates and corresponding 95% confidence intervals (CI) of generalized linear mixed models (GLMMs) assessing the effect of distinct factors on the probability of activity of tapirs in the two study areas SESC (**left** panel) and FBA (**right** panel). (**A**,**B**): probability of nocturnal activity (nocturnal: 18:00–05:59 h, diurnal: 6:00–17:59 h), (**C**,**D**): probability of high nocturnal activity strict (nocturnal records separated into hours with high and low activity), (**E**,**F**): probability of high nocturnal activity lax (crepuscular and nocturnal records (17:00–06:59 h) separated into hours with high and low activity). Factors included in the analysis: habitat at camera trap site (forest, savanna); cattle presence at camera trap site (no, yes); period during the camera trap sample (dry, rainy); and distance of camera trap site to the next freshwater lake, settlement, saltwater lake (only FBA), or dirt roads or trails used for tourism. Random effects, camera trap site ID and sample ID were included in all models.

For SESC, two models were averaged for nocturnal probability, seven models for strict high nocturnal probability and eight models for lax high nocturnal activity. Nocturnal probability was best described by three factors and strict and lax high nocturnal probability were best described by five factors each (Table 2). The results suggest that only nocturnal probability was significantly affected, with higher nocturnal or lower diurnal probability at sites with cattle and during the rainy period (Figure 4, Table S2).

For FBA, 6 models were averaged for nocturnal probability, 12 models for strict high nocturnal probability, and 14 models for lax high nocturnal probability. Nocturnal probability was best described by four factors, and strict and lax high nocturnal probability were best described by five factors each (Table 2). The results suggest that neither the nocturnal nor strict or lax high nocturnal probability was affected by any of the factors (Figure 4, Table S2).

The VIFs of the fitted full models ranged between 1.14 (SE = 1.07) and 3.29 (SE = 1.81) for SESC and 1.05 (SE = 1.02) and 2.02 (SE = 1.42) for FBA, suggesting low correlation among factors.

## 4. Discussion

In accordance with previous studies, lowland tapirs in both areas are mostly nocturnal, stay active all night and show a bimodal activity pattern with two peaks of activity after sunset and before sunrise, which illustrates the species movement between resting and foraging places [57,70,106,107,108]. Previous studies report activity peaks between 19 and 20 pm, 3 and 4 am [106], 20 and 21 pm, 5 and 6 am [32], 19 and 0 pm, 3 and 7 am [107], 20 and 22 pm and 3 and 4 am [108], suggesting a quite similar pattern as during the present study and only little variation among different regions. 

It is important to mention, however, that results from different studies should be compared with caution, as different time-of-independence filters can lead to differences in the estimated activity [109]. Additionally, the identification of different individuals on consecutive images within the 30-minute interval used to determine an independent record might bias the comparison between the two study areas. A successful identification depended on the quality of the record and was thus not always possible. As the individual identification led to only 18 (5%) (SESC) and 19 (6%) (FBA) additional records that would not have been counted following only the 30-minute criterion, and these records were distributed across different camera trap sites and different field surveys, including them might not be a major issue.

When comparing the results among the two study areas, the differences concerning the intensity of land management, flooding regimes and available habitats appear not to cause detrimental changes in the tapirs’ general activity pattern. Additionally, the activity levels were similar, which might indicate comparable foraging effort, movement patterns or exposure to disturbance in both study areas. The results of the GLMMS, however, suggest that there are some differences in the species temporal and spatial responses to human disturbance and environmental factors.

The results of the model selection indicate that while all factors were deemed important to describe variation in number of tapirs in both study areas, only a subset of factors was relevant to determine the species activity. This stresses the need to address the distinct effect of factors on the spatial and temporal response separately. Where spatial adaptation to a factor is observed, temporal adaptations to the same factor might not be needed, affecting the ability to predict activity from this factor. Conversely, temporal adaptation might affect the ability to predict the spatial behavior.

### 4.1. Tourism

Previous studies on the potential impacts of tourism suggest that the numbers of lowland tapirs at camera trap sites decrease with rising numbers of tourists [13], and human activities along trails can interfere with species trail use [71]. In contrast, low-intensity ecotourism reserves can be important refugees for lowland tapirs and other mammals [110]. In the Pantanal, ecotourism has been evidenced to not affect species abundance [29]. The results of the current study support these findings, as no behavioral response toward tourism in either area was observed.

The management approaches for tourism activities in both study areas seem to provide a rather undisturbed environment for the species. Self-implemented regulations on time and number of visitors and limited access to the area might principally reduce the pressure, especially on a nocturnal species. There could be short-term responses to tourism, such as entering dense vegetation when disturbed and returning shortly after, but these short-term effects would be difficult to catch using camera traps [71,111]. The absence of a response could also be related to habituation processes, as tourism has been conducted for more than a decade in both areas. Long-run habituation might negatively impact population fitness by reducing anti-predator responses [112,113], which need to be evaluated separately.

### 4.2. Settlements

Lowland tapirs have been shown to be much more abundant in strictly protected areas than in multiple-use protected areas with settlements and some level of land conversion [114]. Campos [115] reports that there is a negative association between human settlements and lowland tapir detection, suggesting that the species increases its shyness around human core areas. The results by Licona et al. [69] show that within protected areas, proximity to settlements was the only relevant factor for reducing lowland tapirs’ occurrence. Similar results were observed only at SESC, suggesting that the species in this area might be more sensitive toward areas where human activities are concentrated.

Resource availability around settlements also appears to be an important factor influencing the species tolerance towards those areas. Flores et al. [116] observed that during dry periods, when water becomes scarcer, tapirs came closer to settlements or roads. Rivera et al. [117] suggest that lowland tapirs increase habitat use closer to small settlements, presumably to use open areas and secondary forests or reduce predation risk. At FBA, fruit trees such as mango or papaya were actively planted around houses and were observed to attract tapirs as well as many other mammal species as a food source during the fruiting season (authors observation).

### 4.3. Cattle

Studies from the Pantanal have shown that cattle can negatively affect the forest understory, regeneration of plants and fruiting tree diversity and availability [30,45,118,119,120]. Especially during the dry period, when food availability is lowest [45], cattle can increase their use of forage sources that are also important for lowland tapirs [119]. According to previous studies, however, the effect of cattle on the lowland tapir appears not to be pronounced. Results by Eaton et al. [30] suggest that lowland tapirs in the Pantanal are little affected by interference from cattle and related forest vegetation alterations, and might be able to use alternative forage sources in times with cattle-driven fruit depletion. Burs et al. [29] report that the species in the Pantanal is comparably abundant in areas with and without cattle. The results of the current study support these findings. Studies from the Atlantic Forest, however, report that lowland tapirs use areas with cattle ranching mainly for transition between forest patches or when feeding on forest edge vegetation, rather than foraging or resting in these areas [57,115]. This might indicate that a similar result at sites with cattle is mainly related to movement behavior.

As suggested by Ferreira et al. [22], a lack of spatial or temporal response could indicate that adaptive strategies are simply not effective in avoiding disturbance. This could be the case in areas with higher livestock densities. Lowland tapirs require quite large home ranges, with sizes varying between 1 km² and 29.7 km² [31]; thus, avoiding cattle areas might not always be an option. A shift toward less diurnal and more nocturnal activity at sites without cattle, as observed at SESC, might be an effective strategy to avoid disturbance by a few cattle (and associated human activity) and to increase the use of sites during the main activity phase. Similar responses to cattle have been observed for herbivore species on grasslands in Argentina [20].

It is also possible that tapir populations at a cattle-ranching farm are used to sharing the area with cattle, while they might be less tolerant toward cattle at SESC, where they were removed from the wider area in 1998 and only occasionally enter.

### 4.4. Habitat

The results by Desbiez et al. [64] from the southern Pantanal indicate that the species selects various habitat types, including open grassland, scrub grassland, scrub forest and semideciduous forest, but are more abundant in forests. In the northern Pantanal, Cordeiro [63] observed higher tapir densities in forest formations than in grassland formations and a strong preference for Acuri palm forest. In contrast, results by Regolin et al. [67] suggest that the habitat use of tapirs in the southern Pantanal is not affected by the extent of forests, Cerrado cover or habitat characteristics such as the number of Acuri palms. Similarly, the results by Burs et al. [29] indicate no preference for neither closed habitats, particularly dense shrublands and riverine and semideciduous forests, nor open habitats, such as pastures, savannas and grasslands. The results of this study support that tapirs’ habitat use in the southern Pantanal might be quite flexible, while tapirs in the northern Pantanal prefer forest. 

The observed differences in habitat use in the two study areas might be related to the distinction in habitat structure and distribution of water sources. The SESC area generally holds a lower portion of water sources, and forest areas are large and interrupted by large areas of savanna. The FBA area is a dense habitat mosaic of small forests interrupted by patches of savanna and a high number of lakes. Tapirs at FBA might thus more often pass through savanna habitat between forest patches. 

### 4.5. Period

Results of the present study suggest no variation in number of tapirs according to period in both study areas. Given the semiaquatic nature of the species [55], tapirs are well adapted to seasonal changes, and their use of the area appears not to be flood-mediated, as observed for other mammals [121,122]. Elevated forest areas have been shown to act as an important refuge and forage source during the rainy period [123,124], but previous studies suggest that tapirs show only little seasonal variation in habitat use. When seasonal differences in behavior were observed, they were related to the different availability of fruits [57,67,74,75].

Lowland tapirs responded similarly in both study areas, suggesting that lowland tapirs are well-adapted to the differences in flooding regimes and proportions of floodable habitats. At SESC, however, the species shifted activity according to period. During the rainy period in the Pantanal, temperatures increase; thus, the species might avoid the hottest hours of the day by adapting to more nocturnal behavior. A similar pattern was observed by Foerster and Vaughan [125] and Medici [57]. According to the results of Ayala [72], an increase in nocturnal activity could also be related to higher resource availability during this period. At FBA, this behavior was not observed, which might be related to the higher availability of water sources in the area.

### 4.6. Water

Access to permanent water bodies has been shown to be an important requirement for tapirs. For instance, studies from the Atlantic Forest suggest that habitats with direct access to a water source were preferred all year round [57], and distance to the next water body has been shown to be positively related to the occurrence of tapirs [126]. Water bodies are important for thermoregulation; as resting places, defecation sites and for avoidance of ectoparasites; and facilitate movement among forage areas [56,125,126,127]. Tapirs have also been shown to enter water during escape from predators [128]. In addition, as the species has been shown to regularly occur around natural salt licks and to walk several kilometers to get there [87,106,129], the saltwater lakes in the southern Pantanal might provide an important mineral source for tapirs. In the two study areas, distance to freshwater bodies or distance to saltwater lakes at FBA had no effect on number or activity of tapirs, suggesting similar results as those observed in a previous study by Regolin et al. [67]. Responses and adaptations might not be visible in these areas, as permanent water bodies are very abundant, especially at FBA, and thus might not be a limiting factor for lowland tapirs there.

## 5. Conclusions

The present study provides only limited evidence that anthropogenic pressure affects the temporal or spatial pattern of lowland tapirs in the rather pristine Pantanal. Nonetheless, the results stress the need to address both adaptive strategies to obtain a clear picture of the species response. Where spatial adaptations were made, temporal shifts in activity might not be needed. Conversely, temporal adaptations can be an effective strategy to use anthropogenic areas.

Recent research suggests that tapirs show little plasticity to alter their behavior, with similar activity patterns [130] or movement patterns [31] in areas under different human disturbances. Whether the species needs to tolerate human activities or can adapt avoidance strategies seems to depend on the intensity of human use. In areas under lower pressure, adaptive strategies appear to be more likely than in areas with a regular presence of humans and livestock. It is possible that adaptations are more effective in areas with larger undisturbed alternatives. Additionally, in areas with higher anthropological activity, a permanent temporal or spatial avoidance of encounters might limit foraging and resting times to such an extent that the disadvantages of changing behavior might be higher than the advantages. Another reason for the lack of adaptation strategies in the more intensively used study area (FBA) could be a higher habituation to human presence. Such an absence of response toward humans could potentially pose a risk to tapir populations, probably not so much in the well-preserved region where FBA is located, but in other parts of their distribution: lowland tapirs have large home range sizes and walk up to 11 km per day [31]. Thus, they roam across areas with different levels of protection, from areas with wildlife-friendly eco-tourists to farmland where poaching might still occur or roads and traffic are present, and being less shy can therefore be dangerous.

In summary, traditional cattle ranching practices with sufficient access to forest patches within pastures, as well as small-scale tourism based on wildlife observations have no local detrimental consequences for the species. For a successful coexistence and for keeping the Pantanal the stronghold for tapirs it has been until now, these areas have to be large and local communities have to be wildlife-positive.

## Figures and Tables

**Table 1:** Top-ranking generalized linear mixed models (GLMMs) with ≤2 AIC value assessing the effect of distinct factors on number of tapirs in the two study areas SESC and FBA. Models were ranked using Akaike’s Information Criterion (AIC); ΔAIC and AIC weight were calculated from AIC; k = number of parameters. Factors included in the analysis: hab (habitat at camera trap site, forest or savanna), catt (cattle presence at camera trap site, yes or no), per (period during the sample, dry or rainy), sett, tour, fresh, salt (distance of camera trap site to the next settlement, dirt road or trail used for tourism, freshwater lake or saltwater lake (only FBA)); random effects, camera trap site ID (siteID) and sample ID (smpID) were included in all models.

Model	AIC	ΔAIC	AIC Weight	Log Likelihood	k
SESC					
hab + sett + tour + fresh + (1|siteID) + (1|smpID)	2202.5	0	0.13	−1093.24	8
hab + sett + tour + (1|siteID) + (1|smpID)	2202.9	0.46	0.1	−1094.47	7
hab + sett + fresh + (1|siteID) + (1|smpID)	2203	0.51	0.1	−1094.49	7
hab + sett + (1|siteID) + (1|smpID)	2204.1	1.66	0.06	−1096.07	6
hab + per + sett + tour + fresh + (1|siteID) + (1|smpID)	2204.2	1.75	0.05	−1093.11	9
catt + hab + sett + tour + fresh + (1|siteID) + (1|smpID)	2204.5	1.99	0.05	−1093.24	9
FBA					
catt + hab + (1|siteID) + (1|smpID)	2196.3	0	0.06	−1092.16	6
(1|siteID) + (1|smpID)	2196.6	0.33	0.05	−1094.33	4
catt + (1|siteID) + (1|smpID)	2197.3	1.01	0.03	−1093.66	5
catt + hab + per + (1|siteID) + (1|smpID)	2197.6	1.33	0.03	−1091.82	7
per + (1|siteID) + (1|smpID)	2197.7	1.4	0.03	−1093.86	5
sett + (1|siteID) + (1|smpID)	2197.8	1.47	0.03	−1093.89	5
catt + hab + sett + (1|siteID) + (1|smpID)	2198	1.72	0.02	−1092.02	7
hab + (1|siteID) + (1|smpID)	2198.1	1.83	0.02	−1094.07	5
catt + hab + tour + (1|siteID) + (1|smpID)	2198.2	1.86	0.02	−1092.09	7
catt + hab + salt + (1|siteID) + (1|smpID)	2198.2	1.91	0.02	−1092.11	7
catt + hab + fresh + (1|siteID) + (1|smpID)	2198.3	1.98	0.02	−1092.15	7

**Table 2:** Top-ranking generalized linear mixed models (GLMMs) with ≤2 AIC value assessing the effect of distinct factors on the probability of activity of tapirs in the two study areas SESC and FBA. (A) Probability of nocturnal activity (nocturnal: 18:00–05:59 h, diurnal: 6:00–17:59 h), (B) probability of high nocturnal activity strict (nocturnal records separated into hours with high and low activity), and (C) probability of high nocturnal activity lax (crepuscular and nocturnal records (17:00–06:59 h) separated into hours with high and low activity). Models were ranked using Akaike’s Information Criterion for small sample sizes (AICc); ΔAICc and AICc weight were calculated from AICc; k = number of parameters. Factors included in the analysis: hab (habitat at camera trap site, forest or savanna), catt (cattle presence at camera trap site, yes or no), per (period during the camera trap sample, dry or rainy), sett, tour, fresh, salt (distance of camera trap site to the next settlement, dirt road or trail used for tourism, freshwater lake or saltwater lake (only FBA)); random effects, camera trap site ID (siteID) and sample ID (smpID) were included in all models.

Model	AICc	ΔAICc	AICc Weight	Log Likelihood	k
(A)					
SESC					
catt + per + (1|siteID) + (1|smpID)	262.6	0	0.26	−126.19	5
catt + per + fresh + (1|siteID) + (1|smpID)	264.4	1.79	0.11	−126.05	6
FBA					
(1|siteID) + (1|smpID)	230.3	0	0.07	−112.11	3
hab + (1|siteID) + (1|smpID)	231	0.75	0.05	−111.46	4
catt + (1|siteID) + (1|smpID)	231.3	1	0.04	−111.58	4
per + (1|siteID) + (1|smpID)	231.5	1.2	0.04	−111.68	4
hab + per + (1|siteID) + (1|smpID)	231.9	1.59	0.03	−110.85	5
fresh + (1|siteID) + (1|smpID)	232.3	1.97	0.03	−112.07	4
(B)					
SESC					
sett + tour + (1|siteID) + (1|smpID)	384.2	0	0.09	−187	5
(1|siteID) + (1|smpID)	385.2	1.03	0.06	−189.58	3
catt + sett + tour + (1|siteID) + (1|smpID)	385.5	1.23	0.05	−186.57	6
sett + tour + fresh + (1|siteID) + (1|smpID)	385.5	1.23	0.05	−186.57	6
per + sett + tour + (1|siteID) + (1|smpID)	386	1.76	0.04	−186.84	6
sett + (1|siteID) + (1|smpID)	386	1.82	0.04	−188.95	4
sett + fresh + (1|siteID) + (1|smpID)	386.1	1.88	0.04	−187.94	5
FBA					
(1|siteID) + (1|smpID)	367.3	0	0.04	−180.59	3
catt + hab + (1|siteID) + (1|smpID)	367.3	0.07	0.04	−178.55	5
catt + (1|siteID) + (1|smpID)	367.4	0.1	0.04	−179.6	4
catt + sett + (1|siteID) + (1|smpID)	367.4	0.12	0.04	−178.57	5
catt + hab + sett + (1|siteID) + (1|smpID)	367.7	0.47	0.03	−177.7	6
tour + (1|siteID) + (1|smpID)	368	0.73	0.03	−179.92	4
catt + tour + (1|siteID) + (1|smpID)	368.1	0.8	0.03	−178.92	5
catt + hab + tour + (1|siteID) + (1|smpID)	368.2	0.9	0.03	−177.92	6
sett + (1|siteID) + (1|smpID)	368.7	1.4	0.02	−180.26	4
catt + sett + tour + (1|siteID) + (1|smpID)	368.9	1.65	0.02	−178.3	6
salt + sett + (1|siteID) + (1|smpID)	369	1.76	0.02	−178.35	6
hab + (1|siteID) + (1|smpID)	369.3	2	0.02	−180.55	4
(C)					
SESC					
hab + per + (1|siteID) + (1|smpID)	414.1	0	0.09	−201.97	5
catt + per + (1|siteID) + (1|smpID)	415.1	0.97	0.05	−202.46	5
per + (1|siteID) + (1|smpID)	415.1	0.97	0.05	−203.49	4
(1|siteID) + (1|smpID)	415.4	1.3	0.05	−204.68	3
per + sett + (1|siteID) + (1|smpID)	415.5	1.4	0.04	−202.67	5
hab + per + fresh + (1|siteID) + (1|smpID)	415.8	1.61	0.04	−201.74	6
hab + per + sett + (1|siteID) + (1|smpID)	415.9	1.76	0.04	−201.81	6
catt + hab + per + (1|siteID) + (1|smpID)	416	1.84	0.03	−201.85	6
FBA					
(1|siteID) + (1|smpID)	407.9	0	0.05	−200.9	3
per + (1|siteID) + (1|smpID)	408.4	0.48	0.04	−200.12	4
sett + (1|siteID) + (1|smpID)	409	1.14	0.03	−200.45	4
tour + (1|siteID) + (1|smpID)	409.1	1.17	0.03	−200.46	4
catt + hab + (1|siteID) + (1|smpID)	409.1	1.17	0.03	−199.43	5
catt + (1|siteID) + (1|smpID)	409.1	1.23	0.03	−200.49	4
catt + hab + per + (1|siteID) + (1|smpID)	409.2	1.28	0.02	−198.44	6
catt + sett + (1|siteID) + (1|smpID)	409.3	1.39	0.02	−199.53	5
hab + (1|siteID) + (1|smpID)	409.6	1.67	0.02	−200.71	4
per + tour + (1|siteID) + (1|smpID)	409.6	1.69	0.02	−199.69	5
catt + per + (1|siteID) + (1|smpID)	409.6	1.7	0.02	−199.69	5
catt + hab + sett + (1|siteID) + (1|smpID)	409.6	1.73	0.02	−198.66	6
fresh + (1|siteID) + (1|smpID)	409.7	1.76	0.02	−200.76	4
per + sett + (1|siteID) + (1|smpID)	409.7	1.8	0.02	−199.74	5

## Data Availability

Camera trap data are available from https://cobra.ic.ufmt.br/ (accessed on 22 December 2022).

## Supplementary Materials

The following are available online at https://www.mdpi.com/article/10.3390/life13010066/s1, Table S1: Averaged parameter estimates, standard error (SE) and 95% confidence intervals (CI) of generalized linear mixed models (GLMMS) assessing the effect of distinct factors on the number of tapirs in the two study areas SESC and FBA., Table S2: Averaged parameter estimates, standard error (SE) and 95% confidence intervals (CI) of generalized linear mixed models (GLMMS) assessing the effect of distinct factors on the probability of (a) nocturnal activity (18:00–05:59 h), (b) high nocturnal activity strict (18:00–05:59 h) and (c) high nocturnal activity lax (17:00–06:59 h) of tapirs in the two study areas SESC and FBA; Figure S1: Camera trap records of *Tapirus terrestris*.

## References

[1] Dirzo R., Young H.S., Galetti M., Ceballos G., Isaac N.J., Collen B. (2014). Defaunation in the Anthropocene. Science.

[2] Newbold T., Hudson L.N., Hill S.L., Contu S., Lysenko I., Senior R.A., Börger L., Bennett D.J., Choimes A., Collen B. (2015). Global effects of land use on local terrestrial biodiversity. Nature.

[3] Tucker M.A., Böhning-Gaese K., Fagan W.F., Fryxell J.M., Van Moorter B., Alberts S.C., Ali A.H., Allen A.M., Attias N., Avgar T. (2018). Moving in the Anthropocene: Global reductions in terrestrial mammalian movements. Science.

[4] Vié J.C., Hilton-Taylor C., Stuart S.N. (2009). The 2008 Review of the IUCN Red List of Threatened Species.

[5] Quintana R.D. (2003). Seasonal effects on overlap trophic niche between capybara and livestock, and on throphic niche breadths in a rangeland of Central Entre Rios, Argentina. Mammalia.

[6] Chaikina N.A., Ruckstuhl K.E. (2006). The Effect of Cattle Grazing on Native Ungulates: The Good, the Bad, and the Ugly. Rangelands.

[7] Vila A.R., Beade M.S., Barrios Lamunière D. (2008). Home range and habitat selection of pampas deer. J. Zool..

[8] Kinnaird M.F., O’brien T.G. (2012). Effects of Private-Land Use, Livestock Management, and Human Tolerance on Diversity, Distribution, and Abundance of Large African Mammals. Conserv. Biol..

[9] Higginbottom K. (2004). Wildlife Tourism: Impacts, Management and Planning.

[10] Krüger O. (2005). The role of ecotourism in conservation: Panacea or Pandora’s box?. Biodivers. Conserv..

[11] Buckley R.C., Morrison C., Castley J.G. (2016). Net Effects of Ecotourism on Threatened Species Survival. PLoS ONE.

[12] Oberosler V., Groff C., Iemma A., Pedrini P., Rovero F. (2017). The influence of human disturbance on occupancy and activity patterns of mammals in the Italian Alps from systematic camera trapping. Mamm. Biol..

[13] Ouboter D.A., Kadosoe V.S., Ouboter P.E. (2021). Impact of ecotourism on abundance, diversity and activity patterns of medium-large terrestrial mammals at Brownsberg Nature Park, Suriname. PLoS ONE.

[14] Gaynor K.M., Hojnowski C.E., Carter N., Brashares J.S. (2018). The influence of human disturbance on wildlife nocturnality. Science.

[15] Bateman P.W., Fleming P.A. (2012). Big city life: Carnivores in urban environments. J. Zool..

[16] Selier J., Slotow R., Di Minin E. (2015). Large Mammal Distribution in a Transfrontier Landscape: Trade-offs Between Resource Availability and Human Disturbance. Biotropica.

[17] Di Bitetti M.S., Paviolo A., Ferrari C.A., De Angelo C., Di Blanco Y. (2008). Differential responses to hunting in two sympatric species of brocket deer (*Mazama americana* and *M. nana*). Biotropica.

[18] Cruz P., Iezzi M.E., De Angelo C., Varela D., Di Bitetti M.S., Paviolo A. (2018). Effects of human impacts on habitat use, activity patterns and ecological relationships among medium and small felids of the Atlantic Forest. PLoS ONE.

[19] Massara R.L., Paschoal A.M.D.O., Bailey L.L., Doherty P.F., Barreto M.D.F., Chiarello A.G. (2018). Effect ofhumans and pumas on the temporal activity of ocelots inprotected areas of Atlantic Forest. Mamm. Biol..

[20] Di Bitetti M.S., Iezzi M.E., Cruz P., Varela D., de Angelo C. (2020). Effects of cattle on habitat use and diel activity of large native herbivores in a South American rangeland. J. Nat. Conserv..

[21] Pardo L.E., Edwards W., Campbell M.J., Gómez-Valencia B., Clements G.R., Laurance W.F. (2021). Effects of oil palmand human presence on activity patterns of terrestrial mammals in the Colombian Llanos. Mamm. Biol..

[22] Ferreira G.B., Newbold T., Oliveira M.J.R., Pringle H., Pinheiro M.S., de Pinho F.F., Carbone C., Rowcliffe M. (2022). Limited temporal response of Cerrado mammals to anthropogenic pressure in areas under distinct levels of protection. J. Zool..

[23] Nickel B.A., Suraci J.P., Allen M.L., Wilmers C.C. (2020). Human presence and human footprint have non-equivalent effects on wildlife spatiotemporal habitat use. Biol. Conserv..

[24] Li X., Hu W., Bleisch W.V., Li Q., Wang H., Lu W., Sun J., Zhang F., Ti B., Jiang X. (2020). Functional diversity loss and change in nocturnal behavior of mammals under anthropogenic disturbance. Conserv. Biol..

[25] Suraci J.P., Gaynor K.M., Allen M.L., Alexander P., Brashares J.S., Cendejas-Zarelli S., Crooks K., Elbroch L.M., Forrester T., Green A.M. (2021). Disturbance type and species life history predict mammal responses to humans. Glob. Chang. Biol..

[26] O’Farrill G., Galetti M., Campos-Arceiz A. (2013). Frugivory and seed dispersal by tapirs: An insight on their ecological role. Integr. Zool..

[27] Varela D., Flesher K., Cartes J.L., de Bustos S., Chalukian S., Ayala G., Richard-Hansen C. (2019). *Tapirus terrestris*. The IUCN Red List of Threatened Species. https://www.sciencedirect.com/science/article/abs/pii/S0169534705003320.

[28] Flesher K.M., Medici E.P. (2022). The distribution and conservation status of *Tapirus terrestris* in the South American Atlantic Forest. Neotrop. Biol. Conserv..

[29] Burs K., Wistuba R., Schuchmann K.-L., Perazzi P.R., Marques M.I. (2020). Response of mammals to ecotourism, cattle farming, and habitat structure in the Northern and Southern Brazilian Pantanal. Mastozool. Neotrop..

[30] Eaton D.P., Keuroghlian A., Santos M.C.A. (2017). Citizen scientists help unravel the nature of cattle impacts on native mammals and birds visiting fruiting trees in Brazil’s southern Pantanal. Biol. Conserv..

[31] Medici E.P., Mezzini S., Fleming C.H., Calabrese J.M., Noonan M.J. (2022). Movement ecology of vulnerable lowland tapirs between areas of varying human disturbance. Mov. Ecol..

[32] Lermen I.S. (2021). Efeitos do Risco de Predação na Ocorrência Local e no Padrão de Atividade de *Tapirus terrestris* no Nordeste do Pantanal, Brasil. Master’s Thesis.

[33] Oliveira Santos L.G.R. (2009). Ecologia e Conservação de Ungulados Florestais em uma Área do Pantanal. Master’s Thesis.

[34] Cordeiro J.L.P., Fragoso J.M.V., Crawshaw D., Oliveira L.F.B. (2016). Lowland tapir distribution and habitat loss in South America. PeerJ.

[35] Ministério do Meio Ambiente www.mma.gov.br/portalbio.

[36] Padovani C.R. (2017). Conversão da Vegetação Natural do Pantanal para Uso Antrópico de 1976 até 2017 e Projeção para 2050.

[37] Thielen D., Ramoni-Perazzi P., Puche M.L., Márquez M., Quintero J.I., Rojas W., Soto-Werschitz A., Thielen K., Nunes A., Libonati R. (2021). The Pantanal under Siege-On the Origin, Dynamics and Forecast of the Megadrought Severely Affecting the Largest Wetland in the World. Water.

[38] Junk W.J., Bayley P.B., Sparks R.E. (1989). The flood pulse concept in river-floodplain systems. Can. J. Fish. Aquat. Sci.

[39] Hamilton S.K., Sippel S.J., Melack M. (1996). Innundation patterns in the Pantanal wetland of South America determined by passive microwave remote sensing. Arch. Hydrobiol..

[40] Hamilton S.K. (1999). Potential effects of a major navigation project (Paraguay-Parana Hidrovia) on inundation in the Pantanal floodplains. Regul. Rivers Res. Manag..

[41] Seidl A.F., De Silva J.D.S.V., Moraes A.S. (2001). Cattle ranching and deforestation in the Brazilian Pantanal. Ecol. Econ..

[42] Santos S.A., Cardoso E.L., Silva R.A., Pellegrin A.O. (2002). Princípios Básicos para a Produção Sustentável de Bovinos de Corte no Pantanal.

[43] Santos S.A., Crispim S.M.A., Comastri Filho J.A., Cardoso E.L. (2004). Princípios de Agroecologia no Manejo das Pastagens Nativas do Pantanal.

[44] Abreu U.G.P., McManus C., Santos S.A. (2010). Cattle ranching, conservation and transhumance in the Brazilian Pantanal. Pastoralism.

[45] Santos S.A., Desbiez A.L.J., Crispim S.M.A., Comastri Filho J.A., Abreu U.G.P., Rodela L.G. (2010). Natural and cultivated pastures and their use by cattle. The Pantanal: Ecology, Biodiversity and Sustainable Management of a Large Neotropical Seasonal Wetland.

[46] Alho C.J.R., Sabino J. (2011). A conservation agenda for the Pantanal’s biodiversity. Braz. J. Biol..

[47] Alho C.J.R., Silva J.S.V. (2012). Effects of severe floods and droughts on wildlife of the Pantanal wetland (Brazil)—A review. Animals.

[48] Padovani C.R., Dacruz M.L.L., Padovani S.L.A.G. (2004). Desmatamento do Pantanal Brasileiro para o Ano 2000. IV Simpósio Sobre Recursos Naturais e Sócio-Econômicos do Pantanal.

[49] Bergier I. (2013). Effects of highland land-use over lowlands of the Brazilian Pantanal. Sci. Total Environ..

[50] Araujo A.G.J., Obregón G.O., Sampaio G., Monteiro A.M.V., da Silva L.T., Soriano B., Padovani C., Rodriguez D.A., Maksic J., Farias J.F.S. (2018). Relationships between variability in precipitation, river levels, and beef cattle production in the Brazilian Pantanal. Wetlands Ecol. Manag..

[51] Thielen D., Schuchmann K.-L., Ramoni-Perazzi P., Marquez M., Rojas W., Quintero J.I., Marques M.I. (2020). Quo vadis Pantanal? Expected precipitation extremes and drought dynamics from changing sea surface temperature. PLoS ONE.

[52] Libonati R., Belém L.B.C., Rodrigues J.A., Santos F.L.M., Sena C.A.P., Pinto M.M., Carvalho I.A. (2021). Sistema ALARMES- Alerta da Área Queimada Pantanal, Situação Final de 2020.

[53] Marengo J.A., Cunha A.P., Cuartas L.A., Leal K.R.D., Broedel E., Seluchi M.E., Michelin C.M., Baião C.F.D.P., Ângulo E.C., Almeida E.K. (2021). Extreme Drought in the Brazilian Pantanal in 2019–2020: Characterization, Causes, and Impacts. Front. Water.

[54] Taber A.B., Chalukian S.C., Altrichter M., Minkowski K., Lizárraga L., Sanderson E., Rumiz D., Ventincinque E., Moraes A., de Angelo C. (2008). Range-Wide Status Analysis of Lowland Tapir (Tapirus terrestris) and White-Lipped Peccary (Tayassu pecari): Final Report.

[55] Bodmer R.E. (1990). Responses of ungulates to seasonal inundations in the amazon floodplain. J. Trop. Ecol..

[56] Padilla M., Dowler R.C. (1994). *Tapirus terrestris*. Mammalian Species. Am. Soc. Mammal..

[57] Medici E.P. (2010). Assessing the Viability of Lowland Tapir Populations in a Fragmented Landscape. Ph.D. Thesis.

[58] Soto Q.G. (2002). Dieta del Tapir *Tapirus terrestris* y su Rol como Dispersor de Semillas en el Chaco (Cerro Cortado), Provincia Cordillera, Santa Cruz, Bolivia. Bachelor’s Thesis.

[59] Olmos O., Brooks D., Bodmer R., Matola S. (1997). Tapirs as seed dispersers and predators. Tapirs: Status Survey and Conservation Action Plan.

[60] Galetti M., Keuroghlian A., Hanada L., Morato M.I. (2001). Frugivory and seed dispersal by the lowland tapir (*Tapirus terrestris*) in southeast Brazil. Biotropica.

[61] Tófoli C.F. (2006). Frugivoria e Dispersão de Sementes por *Tapirus terrestris* (Linnaeus, 1758) na Paisagem Fragmentada do Pontal do Paranapanema, São Paulo. Master’s Thesis.

[62] Ferreguetti A.C., Tomás W.M., Bergallo H.G. (2017). Density, occupancy, and detectability of lowland tapirs, *Tapirus terrestris*, in Vale Natural Reserve, southeastern Brazil. J. Mammal..

[63] Cordeiro J.L.P. (2004). Estrutura e Heterogeneidade da Paisagem de uma Unidade de Conservação no Nordeste do Pantanal (RPPN SESC Pantanal), Mato Grosso, Brasil: Efeitos Sobre a Distribuição e Densidade de Antas (*Tapirus terrestris*) e de Cervos-do-Pantanal (*Blastocerus dichotomus*). Ph.D. Thesis.

[64] Desbiez A.L.J., Bodmer R.E., Aparecida S. (2009). Wildlife habitat selection and sustainable resources management in a Neotropical wetland. Biodivers. Conserv..

[65] Cañas L.F.S. (2010). Uso do Espaço e Atividade de *Tapirus terrestris* em uma Área do Pantanal Sul. Master’s Thesis.

[66] Castro W.J.P. (2015). Probabilidade de Ocupação de Manchas Florestais por Médios e Grandes Mamíferos na Sub-Região da Nhecolândia, Pantanal, Mato Grosso do Sul, Brasil. Master’s Thesis.

[67] Regolin A.L., Oliveira-Santos L.G., Ribeiro M.C., Bailey L.L. (2021). Habitat quality, not habitat amount, drives mammalian habitat use in the Brazilian Pantanal. Landsc. Ecol..

[68] García M.J., Medici E.P., Naranjo E.J., Novarino W., Leonardo R.S. (2012). Distribution, habitat and adaptability of the genus *Tapirus*. Integr. Zool..

[69] Licona M., McCleery R., Collier B., Brightsmith D.J., Lopez R. (2011). Using ungulate occurrence to evaluate community-based conservation within a biosphere reserve model. Anim. Conserv..

[70] Cruz P., Paviolo A., Bó R.F., Thompson J.J., Di Bitetti M.S. (2014). Daily activity patterns and habitat use of the lowland tapir (*Tapirus terrestris*) in the Atlantic Forest. Mamm. Biol..

[71] Blake J.G., Mosquera D., Loiselle B.A., Romo D., Swing K. (2017). Effects of human traffic on use of trails by mammals in lowland forest of eastern Ecuador. Neotrop. Biodivers..

[72] Ayala G.M.C. (2003). Monitoreo de *Tapirus terrestris* en el Izozog (Cerro Cortado) Mediante el Uso de Telemetria como Base para un Plan de Conservación. Master’s Thesis.

[73] Coelho I.P., Oliveira L.F.B. (2008). The importance of natural licks in predicting Lowland Tapir (*Tapirus terrestris*, Linnaeus 1758) occurrence in the Brazilian Pantanal. Tapir Conserv..

[74] Salas L.A., Fuller T.K. (1996). Diet of the lowland tapir (*Tapirus terrestris* L.) in the Tabaro River valley, Southern Venezuela. Can. J. Zool..

[75] Herrera J.C., Taber A.B., Wallace R.B., Painter R.L.E. (1999). Lowland tapir (*Tapirus terrestris*) behavioral ecology in a southern Amazonian tropical forest. Vida Silv. Neotrop..

[76] Talamoni S.A., Assis M.A.C. (2009). Feeding habit of the Brazilian tapir, *Tapirus terrestris* (Perissodactyla: Tapiridae) in a vegetation transition zone in southeastern Brazil. Zool. Curitiba Impresso.

[77] Oliveira-Santos L.G.R., Machado-Filho L.C.P., Tortato M.A., Brusius L. (2010). Influence of extrinsic variables on activity and habitat selection of lowland tapirs (*Tapirus terrestris*) in the coastal sand plain shrub, southern Brazil. Mamm. Biol..

[78] Desbiez A.L.J., Bodmer R.E., Tomas W.M. (2010). Mammalian Densities in a Neotropical Wetland Subject to Extreme Climatic Events. Biotropica.

[79] Burs K. (2011). Ecology and Biodiversity of Terrestrial Mammals. Bachelor's Thesis.

[80] Adamoli J.O. (1982). Pantanal e suas relações fitogeográficas com os cerrados. Discussão sobre o conceito de “Complexo do Pantanal”. Anais do 32o Congresso Nacional de Botânica.

[81] Nunes da Cunha C., Junk W.J., Leitão-Filho H.F. (2007). Woody vegetation in the Pantanal of Mato Grosso, Brazil: A preliminary typology. Amazoniana.

[82] Guerreiro R.L., Bergier I., McGlue M.M., Warren L.V., de Abreu U.G.P., Abrahão J., Assine M.L. (2019). The soda lakes of Nhecolândia: A conservation opportunity for the Pantanal wetlands. PECON.

[83] Goncalves H.C., Mercante M.A., Santos E.T. (2011). Hydrological cycle. Braz. J. Biol..

[84] Evans T.L., Costa M., Tomas W.M., Camilo A.R. (2014). Largescale habitat mapping of the Brazilian Pantanal wetland: A synthetic aperture radar approach. Remote Sens. Environ..

[85] Beyer H.L. (2004). Hawth’s Analysis Tools for ArcGIS. http://www.spatialecology.com/htools.

[86] O’Brien T.G., Kinnaird M.F., Wibisono H.T. (2003). Crouching tigers, hidden prey: Sumatran tiger and prey populations in a tropical forest landscape. Anim. Conserv..

[87] Noss A.J., Cuellar R.L., Barrientos J. (2003). A camera trapping and radio telemetry Study of Lowland Tapir (*Tapirus terrestris*) in bolivian dry forests. Newsl. IUCN/SSC Tapir Spec. Gr..

[88] Montenegro O.L., Fang T., Montenegro O.L., Bodmer R.E. (1999). Observaciones sobre la estructura de una población de tapires (*Tapirus terrestris*) en el sureste de la Amazonía peruana. Manejo y Conservación de Fauna Silvestre en América Latina.

[89] Holden J., Yanuar A., Martyr D.J. (2003). The Asian tapir in Kerinci Seblat National Park, Sumatra: Evidence collected through photo-trapping. Oryx.

[90] Trolle M., Noss A.J., Cordeiro J.L.P., Oliveira L.F.B. (2008). Brazilian Tapir Density in the Pantanal: A Comparison of Systematic Camera-Trapping and Line-Transect Surveys. Biotropica.

[91] Eisenberg J.F., Brooks D.M., Bodmer R.E., Matola S. (1997). Introduction. Tapirs: Status Survey and Conservation Action Plan.

[92] Heckman C.W. (1998). The Pantanal of Poconé.

[93] R Core Team (2019). R: A Language and Environment for Statistical Computing.

[94] Brooks M.E., Kristensen K., van Benthem K.J., Magnusson A., Berg C.W., Nielsen A., Skaug H.J., Maechler M., Bolker B.M. (2017). glmmTMB Balances Speed and Flexibility Among Packages for Zero-inflated Generalized Linear Mixed Modeling. R J..

[95] Grueber C.E., Nakagawa S., Laws R.J., Jamieson I.G. (2011). Multimodel inference in ecology and evolution: Challenges and solutions. J. Evol. Biol..

[96] Lüdecke D., Ben-Shachar M.S., Patil I., Waggoner P., Makowski D. (2021). Performance: An R Package for Assessment, Comparison and Testing of Statistical Models. JOSS.

[97] James G., Witten D., Hastie T., Tibshirani R. (2013). An Introduction to Statistical Learning: With Applications in R.

[98] Burnham K.P., Anderson D.R. (2002). Model Selection and Multimodel Inference: A Practical Information-Theoretic Approach.

[99] (2020). Bartoń K MuMIn: Multi-Model Inference. R Package Version 1.43.17. https://CRAN.R-project.org/package=MuMIn.

[100] (2021). Rowcliffe M Activity: Animal Activity Statistics. R Package Version 1.3.1. https://CRAN.R-project.org/package=activity.

[101] Rowcliffe J.M., Kays R., Kranstauber B., Carbone C., Jansen P.A. (2014). Quantifying levels of animal activity using camera trap data. MME.

[102] Ridout M., Linkie M. (2009). Estimating overlap of daily activity patterns from camera trap data. JABES.

[103] Meredith M., Ridout M. (2014). Overview of the Overlap Package. R Project. https://cran.r-project.org/web/packages/overlap/vignettes/overlap.pdf.

[104] Lund U., Agostinelli C. (2018). CircStats: Circular Statistics, from “Topics in Circular Statistics”. R Package Version 0.2-6. https://CRAN.R-project.org/package=CircStats.

[105] Sugiura N. (1978). Further analysts of the data by akaikes information criterion and the nite corrections. Commun. Stat. B Simul. Comput..

[106] Tobler M.W. (2008). The Ecology of the Lowland Tapir in Madre de Dios, Peru: Using New Technologies to Study Large Rainforest Mammals. Ph.D. Thesis.

[107] Wallace R.B., Ayala G., Viscarra M. (2012). Lowland tapir (*Tapirus terrestris*) distribution, activity patterns and relative abundance in the greater Madidi-Tambopata landscape. Integr. Zool..

[108] Espinosa S., Salvador J. (2017). Hunters’ landscape accessibility and daily activity of ungulates in Yasuní Biosphere Reserve, Ecuador. Therya.

[109] Peral C., Landman M., Kerley G.I.H. (2022). The inappropriate use of time-to-independence biases estimates of activity patterns of free-ranging mammals derived from camera traps. Ecol. Evol..

[110] Salvador S., Clavero M., Leite Pitman R. (2011). Large mammal species richness and habitat use in an upper Amazonian forest used for ecotourism. Mamm. Biol..

[111] Kays R., Parsons A.W., Baker M.C., Kalies E.L., Forrester T., Costello R., Rota C.T., Millspaugh J.J., McShea W.J. (2016). Does hunting or hiking affect wildlife communities in protected areas?. J. Appl. Ecol..

[112] Higham J.E.S., Shelton E.J. (2011). Tourism and wildlife habituation: Reduced population fitness or cessation of impact?. Tour. Manag..

[113] Geffroy B., Samia D.S.M., Bessa E., Blumstein D.T. (2015). How Nature-Based Tourism Might Increase Prey Vulnerability to Predators. Tree.

[114] Ferreira G.B., Collen B., Newbold T., Oliveira M.J.R., Pinheiro M.S., de Pinho F.F., Rowcliffe M., Carbone C. (2020). Strict protected areas are essential for the conservation of larger and threatened mammals in a priority region of the Brazilian Cerrado. Biol. Conserv..

[115] Campos B.M. (2021). Uso de Habitat e Padrões de Atividade da Anta (*Tapirus terrestris*) em um dos Maiores Remanescentes de Mata Atlântica do Brasil. Master’s Thesis.

[116] Pérez Flores J., Weissenberger H., López-Cen A., Calmé S. (2020). Environmental Factors Influencing the Occurrence of Unhealthy Tapirs in the Southern Yucatan Peninsula. Ecohealth.

[117] Rivera L., Martinuzzi S., Politi N., Bardavid S., De Bustos S., Chalukian S., Lizárraga L., Radeloff V., Pidgeon A. (2021). National parks influence habitat use of lowland tapirs in adjacent private lands in the Southern Yungas of Argentina. Oryx.

[118] Nunes A.P., Tomás W.M., Ragusa-Netto J. (2008). Estrutura do Sub-Bosque em Manchas Florestais no Pantanal da Nhecolândia, Mato Grosso do Sul.

[119] Desbiez A.L.J., Santos S.A., Alvarez J.M., Tomas W.M. (2011). Forage use in domestic cattle (*Bos indicus*), capybara (*Hydrochoerus hydrochaeris*) and pampas deer (*Ozotoceros bezoarticus*) in a seasonal Neotropical wetland. Mamm. Biol..

[120] Tomas W.M., Mourão G., Campos Z., Salis S., Santos S.A. (2009). Intervenções Humanas na Paisagem e Nos Habitats do Pantanal.

[121] Alho C.J.R. (2008). Biodiversity of the Pantanal: Response to seasonal flooding regime and to environmental degradation. Braz. J. Biol..

[122] Mamede S.B., Alho C.J.R. (2006). Response of wild mammals to seasonal shrinking-and-expansion of habitats due to flooding regime of the Pantanal, Brazil. Braz. J. Biol..

[123] Santos S.A. (2001). Caracterização dos Recursos Forrageiros Nativos da Sub-Região da Nhecolandia, Pantanal, Mato Grosso do Sul, Brasil. Ph.D. Thesis.

[124] Desbiez A.L.J., Santos S.A., Keuroghlian A., Bodmer R.E. (2009). Niche Partitioning among White-Lipped Peccaries (Tayassu pecari), Collared Peccaries (Pecari tajacu), and Feral Pigs (Sus scrofa). J. Mammal..

[125] Foerster C.R., Vaughan C. (2002). Home range, habitat use, and activity of Baird’s tapir in Costa Rica. Biotropica.

[126] Naranjo E.J. (1995). Abundancia y uso de hábitat del tapir (Tapirus bairdii) en un bosque tropical húmedo de Costa Rica. Vida Silv. Neotrop..

[127] Naranjo E.J. (2009). Ecology and conservation of Baird’s tapir in Mexico. Trop. Conserv. Sci..

[128] Terwilliger V.J. (1978). Natural history of Baird’s tapir on Barro Colorado Island, Panamá Canal Zone. Biotropica.

[129] Tobler M.W., Carrillo-Percasteguia S.E., Powell G. (2009). Habitat use, activity patterns and use of mineral licks by five species of ungulate in south-eastern Peru. J.Trop. Ecol..

[130] Monette V.D., Kelly M.J., Buchholz R. (2020). Human disturbance and the activity patterns and temporal overlap of tapirs and jaguars in reserves of NW Belize. Biotropica.

