# Supplementary material for: Spatial and Temporal Adaptations of Lowland Tapirs (Tapirus terrestris) to Environmental and Anthropogenic Impacts
Source: Life (Basel). 2022 Dec 25;13(1):66. doi: 10.3390/life13010066 (PMC9866631; doi:10.3390/life13010066)
Supplement: Supplementary file 1 [file life-13-00066-s001.zip › supplMat_tables_fig_legends.pdf]

Table S1. Averaged parameter estimates, standard error (SE), and confidence intervals (CIs) of generalized linear mixed models (GLMMS) assessing the effect of distinct factors on number of lowland tapirs in the two study areas SESC and FBA. Factors included in the analysis: habitat at camera trap site (forest, savanna), cattle presence at camera-trap site (no, yes), period during the sample (dry, rainy), distance of camera-trap site to the next freshwater lake, saltwater lake (only FBA), settlement, dirt road or trail used for tourism; random effects camera-trap site and sample were included in all models.

Factor	Estimate	SE	CI
SESC			
(intercept)	-3.537	0.168	(-3.867, -3.208)
habitat savanna	-0.469	0.185	(-0.831, -0.106)
settlement	0.484	0.209	(0.075, 0.893)
tourism	-0.216	0.218	(-0.644, 0.212)
freshwater	-0.202	0.206	(-0.606, 0.202)
period rainy	0.001	0.033	(-0.063, 0.066)
cattle yes	0.001	0.057	(-0.110, 0.112)
FBA			
(intercept)	-3.513	0.143	(-3.795, 3.232)
cattle yes	0.128	0.139	(-0.143, 0.400)
habitat savanna	-0.106	0.126	(-0.353, 0.141)
period rainy	0.017	0.058	(-0.097, 0.131)
settlement	0.011	0.045	(-0.078, 0.099)
tourism	-0.002	0.026	(-0.054, 0.049)
saltwater	0.002	0.024	(-0.046, 0.050)
freshwater	-0.001	0.023	(-0.046, 0.045)

Table S2. Averaged parameter estimates, standard error (SE) and 95% confidence intervals (CI) of generalized linear mixed models (GLMMS) assessing the effect of distinct factors on the probability of (A) nocturnal activity (18:00-05:59 h), (B) high nocturnal activity strict (18:00-05:59 h), and (C) high nocturnal activity lax (17:00-06:59 h) of lowland tapirs in the two study areas SESC and FBA. Factors included in the analysis: habitat at camera trap site (forest, savanna), cattle presence at camera-trap site (no, yes), period during the camera-trap occasion (dry, rainy), distance of camera-trap site to the next freshwater lake, settlement, saltwater lake (only FBA), dirt roads or trails used for tourism; random effects camera-trap site and occasion were included in all models.

(A)			
Factor	Estimate	SE	CI
SESC			
(intercept)	2.771	0.414	(1.957, 3.585)
cattle yes	0.834	0.291	(0.262, 1.405)
period rainy	1.030	0.290	(0.461, 1.60)

freshwater	-0.040	0.154	(-0.344, 0.263)
FBA			
(intercept)	7.640	1.184	(5.311, 9.969)
habitat savanna	0.210	0.447	(-0.667, 1.088)
cattle yes	0.097	0.314	(-0.519, 0.713)
period rainy	0.218	0.590	(-0.941, 1.378)
freshwater	-0.016	0.176	(-0.362, 0.330)
(B)			
Factor	Estimate	SE	CI
SESC			
(intercept)	0.469	0.133	(0.206, 0.731)
settlement	0.358	0.287	(-0.205, 0.921)
tourism	-0.248	0.261	(-0.761, 0.264)
cattle yes	-0.022	0.082	(-0.183, 0.139)
freshwater	-0.036	0.094	(-0.222, 0.149)
period rainy	0.009	0.054	(-0.098, 0.116)
FBA			
(intercept)	-0.179	0.153	(-0.481, 0.123)
cattle yes	-0.214	0.212	(-0.630, 0.202)
habitat savanna	0.072	0.149	(-0.221, 0.365)
settlement	0.070	0.133	(-0.191, 0.332)
tourism	0.043	0.102	(-0.158, 0.244)
saltwater	-0.005	0.040	(-0.084, 0.074)
(C)			
Factor	Estimate	SE	CI
SESC			
(intercept)	0.617	0.157	(0.308, 0.925)
habitat savanna	0.131	0.179	(-0.221, 0.484)
period rainy	0.250	0.174	(-0.092, 0.591)
cattle yes	0.036	0.103	(-0.167, 0.239)
settlement	0.029	0.092	(-0.152, 0.210)
freshwater	-0.010	0.054	(-0.115, 0.095)
FBA			
(intercept)	-0.051	0.154	(-0.354, 0.252)
period rainy	0.067	0.129	(-0.187, 0.321)
settlement	0.036	0.092	(-0.145, 0.217)
tourism	0.015	0.061	(-0.104, 0.135)
cattle yes	-0.085	0.150	(-0.379, 0.210)
habitat savanna	0.051	0.121	(-0.186, 0.288)
freshwater	0.004	0.034	(-0.063, 0.070)

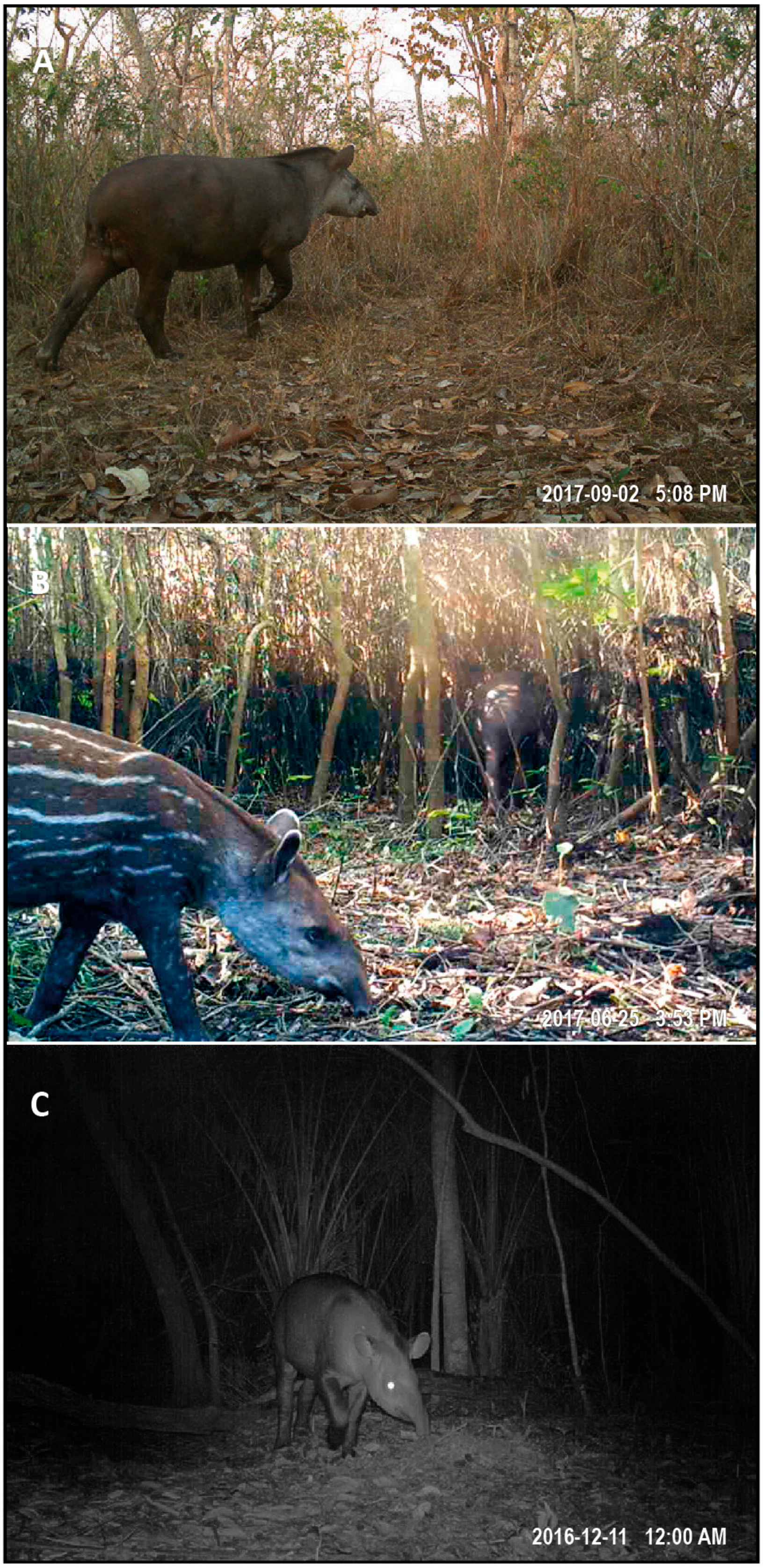

Figure S1. Camera-trap records of *Tapirus terrestris*.
