# Supplementary figures and images for: Spatial and Temporal Adaptations of Lowland Tapirs (Tapirus terrestris) to Environmental and Anthropogenic Impacts
Source: Life (Basel). 2022 Dec 25;13(1):66. doi: 10.3390/life13010066 (PMC9866631; doi:10.3390/life13010066)

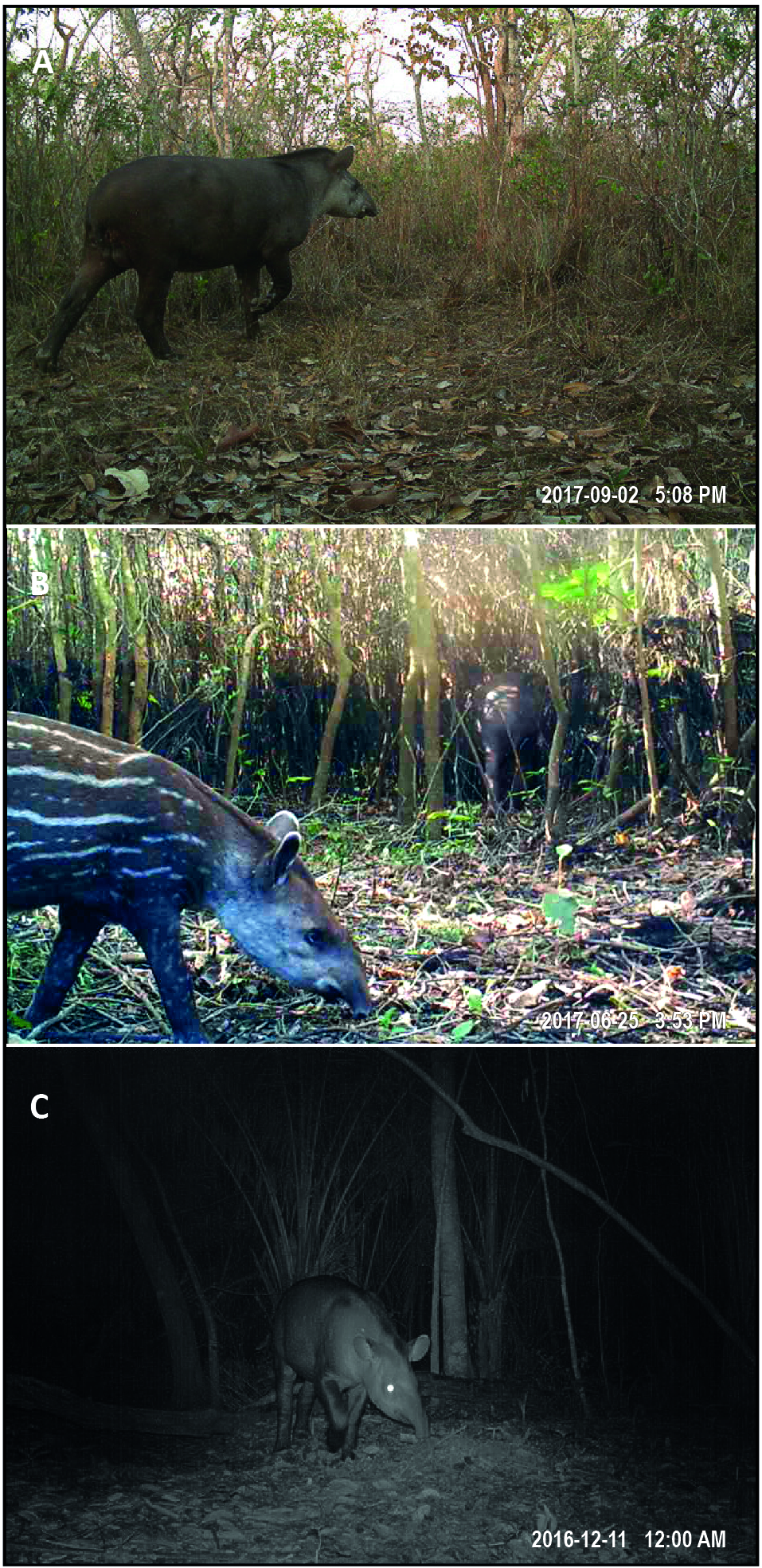

Supplement: Supplementary file 1 [file life-13-00066-s001.zip › FigS1_camera trap records of Tapirus terrestris.jpg]
